# Myeloid-Mediated Immunoregulation and Resistance to Immune Checkpoint Inhibitor Therapy Across Squamous Cell Carcinomas: Mechanisms and Reprogramming Strategies

**DOI:** 10.3390/cancers18172724

**Published:** 2026-08-22

**Authors:** Jaafar A. Hadi, Zohra N. Nizami, Ahmed Tajmim Noor, Abdullah A. Osman, Jeffrey N. Myers

**Affiliations:** 1 Department of Head and Neck Surgery, The University of Texas MD Anderson Cancer Center, Houston, TX 77030, USA; jahadi@mdanderson.org (J.A.H.);; 2 The University of Texas MD Anderson Cancer Center UTHealth Houston Graduate School of Biomedical Sciences, Houston, TX 77030, USA

**Keywords:** squamous cell carcinoma, immune checkpoint inhibitors, tumor immune microenvironment, tumor-associated macrophages, myeloid-derived suppressor cells, dendritic cells, immunotherapy resistance, myeloid reprogramming

## Abstract

Immune checkpoint inhibitors (ICIs) targeting PD-1/PD-L1 have improved outcomes across squamous cell carcinomas (SCCs) of the head and neck, lung, esophagus, and skin, yet durable responses remain confined to a subset of patients in every subtype. Objective response rates vary substantially across SCCs despite overlapping genomic alterations, comparable tumor mutational burden, and high PD-L1 expression, indicating that tumor-intrinsic biomarkers alone do not explain this variability. Growing evidence points to the tumor immune microenvironment, and in particular the myeloid compartment, as a critical determinant of immunotherapy responsiveness. In this review, we synthesize current evidence on myeloid-mediated immune regulation across SCC subtypes, focusing on tumor-associated macrophages, myeloid-derived suppressor cells/tumor-associated neutrophils, and dendritic cells, and the mechanisms by which these populations impair antigen presentation, restrict T cell infiltration, and sustain immunologically “cold” tumor states. We further examine therapeutic strategies aimed at reprogramming rather than simply depleting suppressive myeloid populations, including radiation therapy, STING agonism, and myeloid-targeted agents (CSF1R, PI3Kγ, and CXCR2 inhibition), each of which has shown encouraging preclinical and early clinical activity in combination with ICI. Collectively, this evidence supports a model in which the myeloid compartment functions as an actionable, convergent determinant of ICI resistance across SCC subtypes, rather than merely a passive biomarker. We propose that through the integration of spatial and single-cell profiling of myeloid states with clinical history it will be possible to predict response to immune checkpoint therapy and personalize myeloid-directed combination strategies, though the specific biomarkers needed to match individual patients to a given myeloid-targeted approach remain to be defined. We further discuss the toxicity considerations associated with both immune checkpoint blockade and radiation-based combination approaches, the early-phase status of most myeloid-targeted agents currently in clinical development, and the extent to which mechanistic insight, derived predominantly from HNSCC, generalizes to squamous cell carcinomas arising at other anatomic sites.

## Simple Summary

Immunotherapy drugs known as immune checkpoint inhibitors have improved outcomes for patients with squamous cell carcinomas of the head and neck, lung, esophagus, and skin, yet most patients still do not respond, even when their tumors look similar at the genetic level. Growing evidence points to a group of immune cells called myeloid cells as a key reason why. Tumors actively drive this reprogramming through tumor-derived cytokines, metabolites, and extracellular vesicles, while myeloid cells reciprocally shape tumor cell behavior through bidirectional crosstalk, together establishing a mutually reinforcing immunosuppressive circuit rather than a one-directional or passive process. In this review, we summarize how myeloid cells block anti-tumor immunity across these cancer types and examine emerging strategies, including radiation, drugs that activate innate immune signaling, and agents that target myeloid cells directly, aimed at reprogramming rather than simply eliminating these cells. We also discuss the toxicity, cost, and evidence gaps that must be addressed before these combination strategies can improve outcomes for patients with squamous cell carcinoma.

## 1. Introduction

Squamous cell carcinomas (SCCs) comprise a diverse group of epithelial malignancies arising from stratified squamous epithelia at multiple anatomical sites, including the head and neck, lung, esophagus, and skin. Collectively, these tumors account for a substantial global cancer burden and are frequently associated with chronic inflammatory exposures such as tobacco smoke, alcohol consumption, ultraviolet radiation, and environmental carcinogens [1,2]. Despite their distinct anatomical origins, SCCs share biological and histopathologic characteristics, including squamous differentiation, genomic instability, and inflammatory tumor microenvironments (TMEs) [3,4,5].

Immune checkpoint inhibitors (ICIs) targeting programmed cell death protein-1 (PD-1) and programmed cell death ligand-1 (PD-L1) have emerged as an important therapeutic strategy across several squamous cell carcinoma subtypes. ICIs have improved survival outcomes and produced durable responses in specific subsets of patients with SCC, demonstrating the clinical potential of improving antitumor immunity [6,7,8,9,10,11]. This clinical benefit is accompanied by a distinct toxicity profile: immune-related adverse events (irAEs) occur in an estimated 15–90% of patients depending on agent class, disease setting, and use of combination therapy, with grade 3/4 irAEs reported in approximately 21% of patients across trials; these events can affect the skin, gastrointestinal tract, endocrine organs, liver, and lungs, and occasionally require treatment discontinuation or high-dose immunosuppression [12]. Although we summarize toxicity data for individual agents and combinations above, we have not performed a systematic, cross-trial analysis of combination safety data; the early-phase trials summarized in this review are individually too small and heterogeneous in reporting to support pooled safety estimates, and this represents a genuine gap for the field rather than one this narrative review can close. However, despite these advances, the overall clinical benefit remains limited in a large subset of patients. Objective response rates vary substantially across SCC subtypes, ranging from approximately 15–25% in recurrent or metastatic head and neck SCC (HNSCC) [8,9,11] and esophageal SCC (ESCC) [10] to nearly 50% in advanced cutaneous SCC [6,7], and durable disease control remains limited to a small proportion of treated patients [6,7,8,9,10,11]. Notably, these differences occur despite overlapping genomic alterations, comparable tumor mutational burdens, and high PD-L1 expression, suggesting that conventional tumor-intrinsic biomarkers alone do not fully explain therapeutic response [13,14,15].

Growing evidence indicates that the composition, functional state, and spatial organization of the tumor immune microenvironment may exert a greater influence on immunotherapy outcomes than tumor-intrinsic characteristics alone [15,16]. Although cytotoxic T lymphocytes have traditionally been viewed as the principal mediators of antitumor immunity [17,18], the importance of myeloid populations in regulating antitumor immune responses has become increasingly apparent [19,20]. Advances in single-cell sequencing, spatial transcriptomics, multiplex imaging, and systems immunology have further refined our understanding of myeloid heterogeneity, spatial distribution, and functional states within SCCs [17,18,19,20]. Tumor-associated macrophages (TAMs), myeloid-derived suppressor cells (MDSCs), neutrophils, and dendritic cells, among other myeloid populations, collectively regulate antigen presentation, cytokine production, immune cell trafficking, stromal remodeling, and T cell activation [21,22,23,24]. Consequently, these populations influence not only the baseline immune states but also the development of both primary and acquired resistance to ICI.

Myeloid-mediated immune suppression has emerged as a recurring feature across multiple SCC subtypes. Suppressive myeloid programs have been implicated in impaired antigen presentation, attenuation of type I and type II interferon signaling, recruitment and expansion of regulatory T cells (Tregs), exclusion of cytotoxic T cells, and establishment of immunologically “cold” TMEs [23,24,25,26,27]. Such mechanisms may explain why tumors exhibiting favorable biomarkers, including high PD-L1 expression or high mutational burden, often fail to achieve durable responses to ICIs. Furthermore, differences in myeloid composition and functional polarization may also, in part, account for the marked variability in immunotherapy responsiveness. These observations have generated substantial interest in therapeutic strategies aimed at reshaping the myeloid compartment.

In this review, we examine the emerging roles of myeloid populations as central regulators of ICI responsiveness across SCC subtypes, focusing on HNSCC, LUSC, ESCC, and cSCC, which collectively account for a substantial portion of the global cancer burden [2]. We review the current understanding of the mechanisms through which myeloid populations mediate immune suppression and therapeutic resistance, and highlight emerging strategies aimed at reprogramming the TME through radiation therapy, innate immune activation, and targeted myeloid-directed interventions. While evidence from multiple SCC subtypes identifies shared biological principles, we place particular emphasis on HNSCC, given our group’s longstanding clinical and research focus on this disease. It should also be noted that HNSCC itself is not a single homogeneous entity: the oral cavity, oropharynx, larynx, and hypopharynx differ in HPV association, field cancerization patterns, and lymphatic drainage, and site-specific differences in the immune microenvironment have been described even within HNSCC. The mechanistic evidence synthesized in this review is therefore itself an extrapolation across HNSCC subsites before any extension to other SCC types is considered, and this within-HNSCC heterogeneity should be kept in mind throughout. We propose that the composition, functional state, and spatial organization of the myeloid compartment represent a unifying biological framework that may explain the heterogeneous efficacy of immune checkpoint blockade across SCCs and inform the development of more effective precision immunotherapy strategies. Given the substantial heterogeneity across SCC subtypes in tumor biology, the specific myeloid-targeted agents evaluated, and the design of the trials discussed throughout this review, we present this work as a narrative synthesis of the current evidence rather than a formal meta-analysis; a quantitative pooled analysis would not be methodologically valid across such heterogeneous study designs and endpoints, and we have aimed instead to identify converging mechanistic themes while explicitly flagging where cross-study comparisons are limited by this heterogeneity. To help readers distinguish the strength of evidence underlying different claims in this review, we characterize mechanisms as: (1) well-established, supported by concordant preclinical mechanistic data and human correlative or clinical data (e.g., TAM-mediated T-cell exclusion via PD-L1); (2) preclinically established but clinically unconfirmed, supported by robust mechanistic and animal model data without corresponding human trial validation (e.g., PI3Kγ macrophage reprogramming); or (3) hypothesis-generating, supported by limited, indirect, or cross-tumor-type evidence not yet directly tested in SCC (e.g., the gut microbiome and immunometabolic mechanisms discussed above). We have aimed to flag category (3) claims explicitly with appropriately hedged language throughout, rather than presenting all mechanistic claims with uniform confidence.

## 2. Differential Responses to Immunotherapy Across SCCs

The clinical experience with ICI across SCCs demonstrates that immunotherapy sensitivity is not uniform. We have summarized the major immunotherapy clinical trials across the four major SCC subtypes (HNSCC, LUSC, ESCC, and cSCC) over the past 10–15 years in Table 1. While these trials show that PD-1/PD-L1 blockade improved clinical outcomes in different settings, there are important differences in responses across the SCC subtypes that warrant further investigation.

PD-1 blockade has emerged as an important therapeutic strategy in HNSCC, particularly for recurrent and/or metastatic disease. CheckMate 141 established nivolumab (anti-PD-1) as a survival-improving therapy for platinum-refractory recurrent/metastatic HNSCC, with an objective response rate of 13.3% compared to one of 5.8% with standard single-agent therapy (methotrexate, docetaxel, or cetuximab). However, only a subset of patients achieved durable disease control, and most patients ultimately progressed; in the related KEYNOTE-012 experience with single-agent pembrolizumab in this setting, median duration of response was not reached, but 85% of responses lasted at least 6 months, illustrating that although the proportion of patients who respond to single-agent PD-1 blockade in R/M HNSCC is small, those who do respond can maintain disease control for a prolonged period [28]. KEYNOTE-040 subsequently confirmed the clinical activity of pembrolizumab, another anti-PD-1 antibody, in previously treated recurrent/metastatic HNSCC, although the survival benefit in the overall population was more modest than that observed in another trial, CheckMate 141 [28]. KEYNOTE-048 subsequently moved pembrolizumab (anti-PD-1) into the first-line recurrent/metastatic setting, with monotherapy achieving objective response rates of 23% in patients with a PD-L1 combined positive score (CPS) ≥ 20, 19% in patients with CPS ≥ 1, and 17% in the overall population [8]. Although KEYNOTE-048 established PD-1-based therapy as a first-line standard in recurrent/metastatic HNSCC, objective responses remained limited to a subset of patients, again highlighting that PD-L1 positivity and clinical eligibility for immunotherapy do not reliably translate into durable response.

It is important to note that PD-L1 scoring is not uniform across SCC subtypes: HNSCC and ESCC trials have used the combined positive score (CPS), which incorporates PD-L1 staining on both tumor and infiltrating immune cells, whereas cSCC and squamous NSCLC trials have more commonly relied on the tumor proportion score (TPS), which considers tumor cell staining alone. Because CPS captures PD-L1 expression on tumor-infiltrating myeloid and other immune populations while TPS does not, the two scores are not interchangeable, and cross-subtype comparisons of “PD-L1-high” versus “PD-L1-low” disease should be interpreted with this distinction in mind; direct correlation of CPS versus TPS with myeloid infiltration has not been systematically reported and remains an open question. Consistent with the imperfect predictive value of PD-L1 status, PD-L1-negative or low-CPS tumors still derive some benefit from PD-1 blockade in HNSCC: in KEYNOTE-012, objective response rates were 21.5% in CPS-positive versus 4% in CPS-negative tumors, and in KEYNOTE-055, response rates were 18% in CPS ≥ 1 versus 12% in CPS < 1 tumors, indicating that a subset of PD-L1-negative tumors still respond and that PD-L1 status alone is an imperfect selection biomarker [41,42].

More recently, two phase III trials have extended support for PD1-blockade in the treatment of resectable locally advanced HNSCC. KEYNOTE-689 demonstrated that perioperative pembrolizumab (anti-PD-1) significantly improved event-free survival in the total population (36-month EFS 57.6% versus 46.4%; HR 0.73, 95% CI 0.58–0.92, *p* = 0.008), with a similar magnitude of benefit in the PD-L1 CPS ≥ 1 subgroup (HR 0.70, 95% CI 0.55–0.89) when added to standard-of-care surgery and chemoradiation. NIVOPOSTOP, similarly, showed that the addition of nivolumab to cisplatin-based postoperative chemoradiation improved 3-year disease-free survival in resected, high-risk, locally advanced HNSCC (63.1% versus 52.5%; HR 0.76, 95% CI 0.60–0.98, *p* = 0.034), a benefit that was consistent across PD-L1 CPS subgroups [30]. While these trials do represent breakthroughs for the field, they still highlight a persistent biological problem: a subset of tumors continue to recur, raising the question as to why many tumors fail to generate durable immune-mediated tumor control. These efficacy gains were accompanied by a measurable increase in treatment burden: grade ≥ 3 treatment-related adverse events occurred in 44.6% of patients on the pembrolizumab arm of KEYNOTE-689 versus 42.9% on the control arm, with serious treatment-related adverse events nearly doubled (19.1% versus 10.5%) and grade ≥ 3 immune-related adverse events occurring in 10.0% of pembrolizumab-treated patients. In NIVOPOSTOP, grade 4 adverse events were more frequent with nivolumab plus chemoradiotherapy than with chemoradiotherapy alone during the on-treatment period (13.1% versus 5.6%), although treatment-related deaths were rare and similar between arms [30]. This overlapping toxicity profile is a recurring feature of myeloid- and immune-directed combination strategies more broadly and is discussed further below.

Overlapping toxicity is a realistic concern for these combinations: pexidartinib carries an FDA boxed warning for serious and potentially fatal hepatotoxicity as a class-defining safety signal for CSF1R inhibition [43], and PI3Kγ inhibition with eganelisib has been associated with transaminase elevation and cytopenias in early-phase trials [44]. Whether these signals compound the immune-related adverse events associated with checkpoint blockade has not yet been systematically characterized in randomized combination data.

A similar pattern is observed in LUSC/squamous non-small-cell lung cancer (NSCLC), where checkpoint inhibition is now routinely integrated in advanced and perioperative treatment settings [8,34,35,45]. In CheckMate 017, nivolumab (anti-PD-1) improved overall survival (9.6 months vs. 6 months with docetaxel) and achieved an objective response rate of approximately 20% in advanced squamous-cell NSCLC [34]. In the first-line metastatic setting, KEYNOTE-406 showed that pembrolizumab (anti-PD-1) plus chemotherapy substantially improved objective response rates compared with chemotherapy alone (57.9% vs. 38.4%) in patients with untreated metastatic squamous NSCLC [34,35].

Similarly for ESCC, as demonstrated in ATTRACTION-3, nivolumab (anti-PD-1) improved overall survival compared with taxane chemotherapy in previously treated advanced ESCC, supporting PD-1 blockade as an important therapeutic option in this disease [10]. However, only a subset of patients achieved durable clinical benefit, and most tumors ultimately progressed despite treatment [10]. In the first-line setting, KEYNOTE-590 showed that pembrolizumab plus cisplatin and 5-fluorouracil improved survival compared with chemotherapy alone in advanced ESCC, particularly in patients with PD-L1 CPS ≥ 10 [40]. CheckMate 648 further validated first-line strategies in advanced ESCC, demonstrating improved overall survival with nivolumab plus chemotherapy and with nivolumab plus ipilimumab (anti-CTLA4) compared with chemotherapy alone [39]. These findings indicate that although PD-1 blockade can improve outcomes in ESCC, additional mechanisms of immune resistance continue to limit therapeutic efficacy in many patients.

PD-1 blockade also transformed management of advanced cutaneous squamous cell carcinoma (cSCC). The phase II EMPOWER-CSCC-1 trial established cemiplimab as the standard of care in locally advanced and metastatic cSCC, with an objective response rate of approximately 47% and durable responses maintained at extended follow-up, with an estimated 88.3% of responders remaining in response at 12 months in updated analyses; in the adjuvant setting, C-POST similarly demonstrated that immunotherapy-driven disease control is durable rather than transient, with a 24-month disease-free survival rate of 87.1% for cemiplimab versus 64.1% for placebo [6]. More recently, neoadjuvant cemiplimab has moved PD-1 blockade into the perioperative setting for resectable disease: in a phase II trial of patients with stage II to IV cSCC, neoadjuvant cemiplimab produced a pathologic complete response in 51% of patients and a major pathologic response in an additional 13%, meeting the study’s primary endpoint and enabling function-preserving surgery in a subset of patients [46]. While these studies suggest that cSCC is among the more ICI-responsive SCC subtypes, primary resistance, incomplete pathologic response, and disease recurrence still occur, indicating that even highly immunogenic SCCs can maintain or acquire immune-evasive TME states. While these studies suggest that cSCC is among the more ICI-responsive SCC subtypes, primary resistance, incomplete pathologic response, and disease recurrence still occur, indicating that even highly immunogenic SCCs can maintain or acquire immune-evasive TME states.

An important caveat to this comparison across subtypes is that the trials summarized in Table 1 differ substantially in patient selection, prior treatment exposure, disease setting (recurrent/metastatic versus perioperative), and PD-L1 scoring system, none of which were harmonized across studies; the approximately 15–50% range in objective response rates across HNSCC, ESCC, squamous NSCLC, and cSCC therefore reflects both trial-design heterogeneity and true biological differences between subtypes, and no formal cross-trial statistical test can partition these two sources of variability in the absence of individual patient-level data or a prospective head-to-head comparison. This heterogeneity extends to treatment line and setting: Table 1 spans first-line, second-line-or-later, adjuvant, and neoadjuvant/perioperative trials, and response rates are not directly comparable across these settings. Likewise, although comparable tumor mutational burden and PD-L1 expression have been reported across several SCC subtypes individually, no published dataset to date has jointly assessed tumor mutational burden alongside myeloid compartment composition within the same patient cohort. A further limitation is that tumor mutational burden (TMB) values themselves are not directly comparable across the studies discussed, since TMB is calculated using different sequencing panels, bioinformatic pipelines, and cutoffs across trials, and, as discussed above, PD-L1 is scored using non-interchangeable CPS and TPS systems across tumor types; both factors independently limit the validity of any cross-subtype comparison of response rates against biomarker status, beyond the confounding from differing trial designs already noted. The claim that myeloid programs, rather than tumor-intrinsic biomarkers, account for the residual variability in response should therefore be read as a synthesis of separate lines of evidence rather than a directly demonstrated association. This heterogeneity in treatment setting is substantial and worth making explicit: the trials summarized in Table 1 span the full range of clinical contexts, from second-line-or-later platinum-refractory disease (CheckMate 141, KEYNOTE-040, CheckMate 017, ATTRACTION-3) to first-line recurrent/metastatic disease (KEYNOTE-048, KEYNOTE-407, CheckMate 9LA, IMpower131, KEYNOTE-590, CheckMate 648), neoadjuvant/perioperative treatment of resectable disease (KEYNOTE-689, MATISSE), and adjuvant treatment following definitive local therapy (NIVOPOSTOP, C-POST, and the consolidation setting of PACIFIC). Response rate and survival comparisons across this table should therefore never be read as head-to-head comparisons of drug efficacy; a first-line trial in treatment-naive patients and a second-line-or-later trial in platinum-refractory patients are not comparable populations regardless of the SCC subtype or agent under study, and the apparent differences in outcome between, for example, HNSCC and squamous NSCLC trials in Table 1 partly reflect this distribution of settings rather than a pure subtype-level biological difference.

Taken together, these clinical trials demonstrate that although ICIs have improved clinical outcomes across multiple SCC subtypes (Table 1), durable clinical benefit remains limited to a subset of patients. The critical question is not whether ICI-mediated antitumor immunity is an effective therapeutic strategy, but rather why most patients fail to derive durable benefit. The persistence of therapeutic resistance across both metastatic and perioperative settings, together with the limited predictive capacity of tumor-intrinsic biomarkers, suggests that additional factors influence treatment outcomes. Increasing evidence points to the TME as a central regulator of therapeutic responsiveness, highlighting the need to identify biomarkers that capture the cellular and functional states of the immune landscape and to develop therapeutic strategies capable of overcoming TME-mediated ICI resistance.

## 3. Myeloid Populations in SCC and Mechanisms of Myeloid-Mediated Resistance

**Figure 1:**
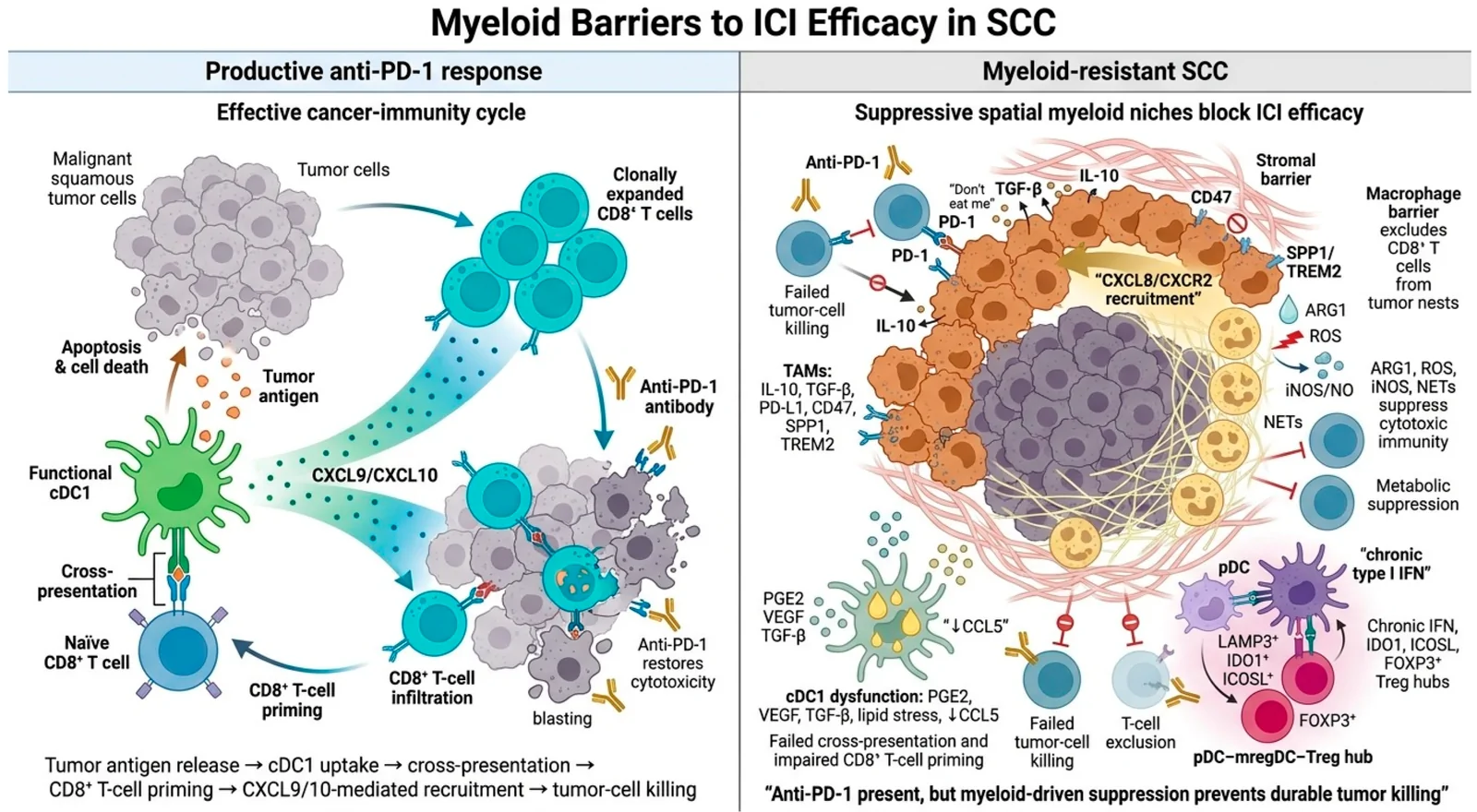
Myeloid barriers to immune checkpoint inhibitor efficacy in squamous cell carcinoma. In responsive tumors (**left**), functional cDC1-mediated cross-presentation primes naïve CD8^+^ T cells, while CXCL9/CXCL10 axis promotes intratumoral infiltration of T cells and facilitates T cell mediated-cytotoxicity following PD-1 blockade. In contrast, resistant SCCs (**right**) contain suppressive myeloid niches in which tumor-associated macrophages (TAMs), PMN-MDSCs/tumor-associated neutrophils (TANs), dysfunctional cDC1s, and pDC/mregDC–Treg cooperatively impair antigen presentation, restrict T cell recruitment and infiltration, impose cytokine- and metabolism-mediated suppression, and establish physical barriers that prevent effective cytotoxic immunity despite anti-PD-1 therapy. Arrows: up, upregulation; down, downregulation.

The myeloid compartment has emerged as a central regulator of antitumor immunity in SCC, capable of shaping both baseline immune states and responses to immunotherapy. As mentioned in the previous section, across the major SCC subtypes, resistance to ICI cannot be explained by checkpoint-ligand expression alone. In this regard, myeloid cells are an important variable as they regulate multiple facets of cancer immunity, including tumor antigen uptake and presentation, T cell priming, local cytotoxic functions, etc. This is especially relevant in SCCs, where ICI failure is seen despite immune infiltration, suggesting a function failure due to the myeloid compartment. In this section, we review the main myeloid populations that play a role in ICI resistance, including tumor-associated macrophages (TAMs), neutrophils, and dendritic cells (Figure 1).

### 3.1. Tumor-Associated Macrophages as Central Drivers of Immune Resistance

Tumor-associated macrophages (TAMs) are the most abundant immune population in SCC TMEs, and have emerged as central orchestrators of tumor-associated inflammation and immune regulation [16]. Classically, macrophages have been described using the M1/M2 polarization paradigm. M1 macrophages are broadly associated with pro-inflammatory and antitumor functions, while M2 macrophages are associated with tissue repair, immune suppression, and tumor progression [47,48,49,50,51]. However, single-cell profiling studies have expanded the functional states of TAMs beyond this dichotomous classification [52,53,54,55]. Rather than discrete M1 and M2 populations, TAMs comprise multiple context-dependent subsets with distinct programs, including antigen presentation, interferon signaling, angiogenesis, lipid metabolism, tissue remodeling, and immune suppression [52,54,55]. This functional diversity is particularly relevant in solid tumors, where TAM populations are shaped by local TME cues and may exert divergent effects on antitumor immunity and therapeutic responsiveness within the same tumor [52,56]. TAM polarization is shaped by tumor-derived cytokines, metabolic stress, hypoxia, and stromal cues. Notably, M2 polarization can be driven by IL-4 and IL-13 through STAT6/PPARgamma signaling [57], TGF-beta/SMAD signaling [58], lactate accumulation, hypoxia-HIF-1alpha/PKM2 activity [59], and tumor-derived Sonic Hedgehog signaling [60].

Once polarized and infiltrated into the TME, TAMs suppress adaptive immunity through multiple mechanisms. TAM-derived IL-10 suppresses antigen presentation and production of inflammatory cytokines, while TAM-derived TGF-β suppresses CD8+ T cell function, promoting cytotoxic T cell exclusion and Treg infiltration [16,61,62], DC cell [16,63]. Furthermore, macrophages also directly engage inhibitory immune receptors; PD-L1 on TAMs directly suppresses PD-1-expressing T cells [64], CD47/SIRPalpha signaling modulates phagocytosis and antigen presentation [65], and VISTA or other myeloid checkpoint molecules can further influence immune suppression [66].

Several TAM subsets appear particularly relevant to ICI resistance in SCCs. In oral SCC, CD163^+^CD204^+^ TAMs produced IL-10 and PD-L1 reducing CD3+ T cell activation in vitro, and negatively correlated with 5-year progression-free survival [67]. This finding is notable because the Kubota et al. cohort predates the era of immune checkpoint inhibitor use in OSCC, supporting that myeloid signatures carry prognostic information even outside the context of immunotherapy, though whether this reflects a shared biology with ICI resistance or a distinct tumor-aggressiveness phenotype has not been formally tested. TREM2+ TAMs (characterized by expression of *C1QA*, *C1QB*, *C1QC*, *TREM2*, *SPP1*, and *APOE*), an emerging TAM subset across cancers, was associated with poor prognosis and reduced sensitivity to anti-PD-1 therapy [68]. In HNSCC, a discrete TAM subset, PF4+ TAMs were associated with decreased response to anti-CD276 therapy through increase in exhausted CXCR6^+^CD8^+^ T cells via the CXCL16-CXCR6 axis [69]. Importantly, spatial organization also plays an important role in molecular suppression. In HNSCC, Naei et al. reported that ICI non-responders exhibit a higher density of localized PD-1/PD-L1 interactions on TAMs, specifically clustered at the tumor-stroma interface to create a functional immunosuppressive barrier [70]. Comprehensive multi-region spatial datasets directly comparing responders and non-responders remain sparse within SCC specifically, and this represents an important limitation of the spatial data currently available. An additional layer of complexity is intratumoral heterogeneity: myeloid density and polarization can vary substantially between regions of the same tumor, and single-region sampling (the norm in most clinical correlative studies to date) may misclassify a tumor’s overall immune state, potentially explaining discordant outcomes among patients nominally sharing the same bulk-level myeloid signature. Beyond the tumor-stroma interface localization already described, myeloid cells occupying distinct spatial niches within the TME likely differ functionally: TIE2+ perivascular macrophages are positioned adjacent to tumor vasculature, where they regulate angiogenesis, transiently increase vascular permeability to promote tumor cell intravasation, and recruit other tumor-promoting leukocytes to the vessel wall, while deeper intratumoral macrophages are more directly positioned to interact with and suppress infiltrating T cells at the site of antigen encounter [71]. Because most correlative studies discussed in this review report bulk or single-region myeloid density without distinguishing perivascular from intratumoral localization, it remains unknown whether the immunosuppressive activity attributed to a given myeloid population reflects its total abundance or specifically its localization relative to tumor vasculature and T-cell infiltration routes, and whether myeloid-directed therapies act preferentially on one spatial niche over the other.

### 3.2. Tumor-Associated Neutrophils and Granulocytic MDSCs as Amplifiers of Immune Suppression

Myeloid-derived suppressor cells (MDSCs) represent another major immunosuppressive component of the myeloid compartment. They arise through pathological myelopoiesis driven by chronic inflammation and cancer, resulting in the accumulation of immature and dysregulated myeloid populations with potent immunosuppressive properties [20,72,73,74]. Throughout this review, and in the broader literature, tumor-associated neutrophils (TANs) and PMN-MDSCs are frequently used interchangeably, but this conflation obscures real biological distinctions. TANs and PMN-MDSCs share overlapping surface markers and cannot currently be reliably distinguished by immunophenotyping alone; however, transcriptomic comparisons indicate that PMN-MDSCs isolated from the same patient occupy a distinct transcriptional state from conventional neutrophils, with the two populations showing genuinely different maturation trajectories rather than representing a single cell type under different names. We use ‘TAN’ here as the broader anatomical/functional descriptor for tumor-infiltrating neutrophil-lineage cells and reserve ‘PMN-MDSC’ specifically for the immunosuppressive, functionally validated subset, while acknowledging that most of the clinical and correlative literature cited throughout this review does not make this distinction rigorously, which limits the precision of the mechanistic claims that can be drawn from it [75]. Mechanistically, these populations impair antigen presentation and T-cell function through several convergent pathways: arginase-1 depletes extracellular L-arginine, which causes rapid downregulation of the T-cell receptor ζ chain (CD3ζ), the principal TCR signal-transduction component, and a corresponding decrease in T-cell proliferation [76]; myeloid-derived reactive oxygen species and peroxynitrite nitrate tyrosine residues on the TCR-CD8 complex itself, disrupting its ability to bind peptide-MHC and rendering CD8+ T cells unresponsive to antigen-specific stimulation despite retaining responsiveness to nonspecific stimuli [77]; and direct PD-L1 expression on myeloid cells engages PD-1 on adjacent T cells, providing an additional, ICI-relevant inhibitory signal independent of tumor cell PD-L1 expression itself, a mechanism directly demonstrated using myeloid-specific PD-L1 knockout mice, in which loss of PD-L1 on tumor-associated macrophages restored intratumoral CD8+ T-cell function [78]. These three mechanisms are not mutually exclusive and likely act in combination within a given suppressive myeloid niche, which may explain why single-pathway interventions have shown limited clinical efficacy relative to their preclinical promise, as discussed throughout the Emerging Myeloid-Targeted Therapeutic Strategies section. Contemporary models classify MDSCs into two principal subsets: polymorphonuclear/granulocytic MDSCs (PMN-MDSCs), which share phenotypic characteristics with tumor-associated neutrophils (TANs), and monocytic MDSCs (M-MDSCs), which resemble activated monocytes, although both populations possess distinct transcriptional, metabolic, and functional programs that differentiate them from their conventional counterparts [72,79]. However, a persistent challenge in the literature is the lack of strict definitions, leading to these terms frequently being used interchangeably. For the purposes of this review, we will focus on neutrophils and PMN-MDSCs, as across various cancers, including SCCs, elevated neutrophil infiltration consistently correlates with poorer survival outcomes [23], drawing significant interest to these populations as integral components of the immune TME.

PMN-MDSCs and TANs suppress antitumor immunity through multiple complementary mechanisms [20,72,80,81,82]. Arginase 1 released from TANs depletes L-arginine, reduces CD3zeta expression, IL-2 production, IFN-gamma secretion, and T cell proliferation [83]. TANs produce reactive oxygen species primarily through NOX2 and STAT3-dependent pathways, which nitrate T cell receptor components and promote antigen-specific tolerance [84,85]. Additionally, iNOS-derived nitric oxide suppresses T cell function by disrupting IL-2 signaling [86].

TAN-mediated immunosuppression and resistance to ICI also relies on local TME cues and post-translational stabilization of inhibitory ligands. Within the tumor niche, intratumoral hypoxia directly upregulates PD-L1 expression on TANs via HIF-signaling, which is further sustained in a IL-10/STAT3 positive feedback loop by infiltrating T cells [87]. Additionally, GPR84, a free fatty acid receptor, enriched on TANs, physically stabilizes surface PD-L1 by restricting endosomal trafficking for lysosomal degradation [88]. This reinforces localized T cell exhaustion, and represents a distinct mechanism of TAN-mediated resistance to ICI.

Another important aspect of TAN-mediated immune suppression is the formation of neutrophil extracellular traps (NETs), which add a spatial dimension. NETs are formed through a specialized process called NETosis, resulting in the release of decondensed DNA, histones, and granular proteins, which form a physical barrier and structural anchor for recruitment of other immune cells [89]. High NET scores have been correlated with poor immunotherapy response in various cancers, including SCCs [90]. IL-8 and other CXCR1/CXCR2 agonists induce NET release from TANs [91], and NETs can limit contact between malignant cells and cytotoxic CD8+ T cells or natural killer cells [92]. Long-term NET exposure has also been reported to increase expression of T cell exhaustion markers such as PD-1, LAG-3, and TIM-3 [93]. Thus, NETs represent physical immune barriers that can prevent the efficacy of ICI even in TMEs with T cell infiltration.

### 3.3. Dendritic Cell Dysfunction Shapes SCC Immune Landscape

DC dysfunction represents another important mechanism of immune resistance by limiting the initiation of productive adaptive immune responses, and acts upstream of T cell exhaustion. DCs are essential for the priming and activation of tumor specific T cells and serve as critical mediators linking innate and adaptive immunity [94,95]. Among dendritic cell subsets, conventional type 1 DC (cDC1s) play a central role in antitumor immunity through their capacity for antigen cross-presentation and CD8+ T cell priming [27,96,97,98,99,100].

Once activated via BATF3 and IRF8, cDC1s promote antitumor immunity by presenting tumor antigen on MHC I, and secreting IL-12 to support cytotoxic T cell differentiation [101] and CXCL9/CXCL10 to recruit CXCR3+ effector T cells [102]. These functions directly relate to ICI response, as effective PD-1 blockade requires tumor-specific T cells that can be primed, expanded, recruited, and maintained. Consequently, cDC1 recruitment is impaired by PGE2 [102], TGF-beta, or IL-10 [103], ICI may fail despite the presence of antigen-specific T cells.

It is important to note that in a suppressive tumor context, both cDC1s and cDC2s can transform to a mature regulatory DC (mregDC) program. After antigen acquisition, both populations can mature into LAMp3+CCR7+ DCs that co-express inhibitory ligands, such as PD-L1, PD-L2, and IDO1 alongside classic maturation markers [104]. Additionally, mregDCs upregulate PD-L1 through AXL signaling. Hence, these DCs do not act as conventional stimulatory APCs, but rather acquire tolerogenic features that drive Treg recruitment and reinforce resistance to ICI. This is also highlighted in an emerging pDC-IFN-mregDC axis. While primary refractory tumors often show structural immune exclusion driven by cancer-associated fibroblasts, tumors with abundant CD8 T cell infiltration can remain resistant due to chronic type I IFN signaling driven by plasmacytoid DCs [105]. This also promotes the expansion of mregDCs and the recruitment of Tregs, resulting in suppressive niches [106].

## 4. Therapeutic Reprogramming Strategies

**Figure 2:**
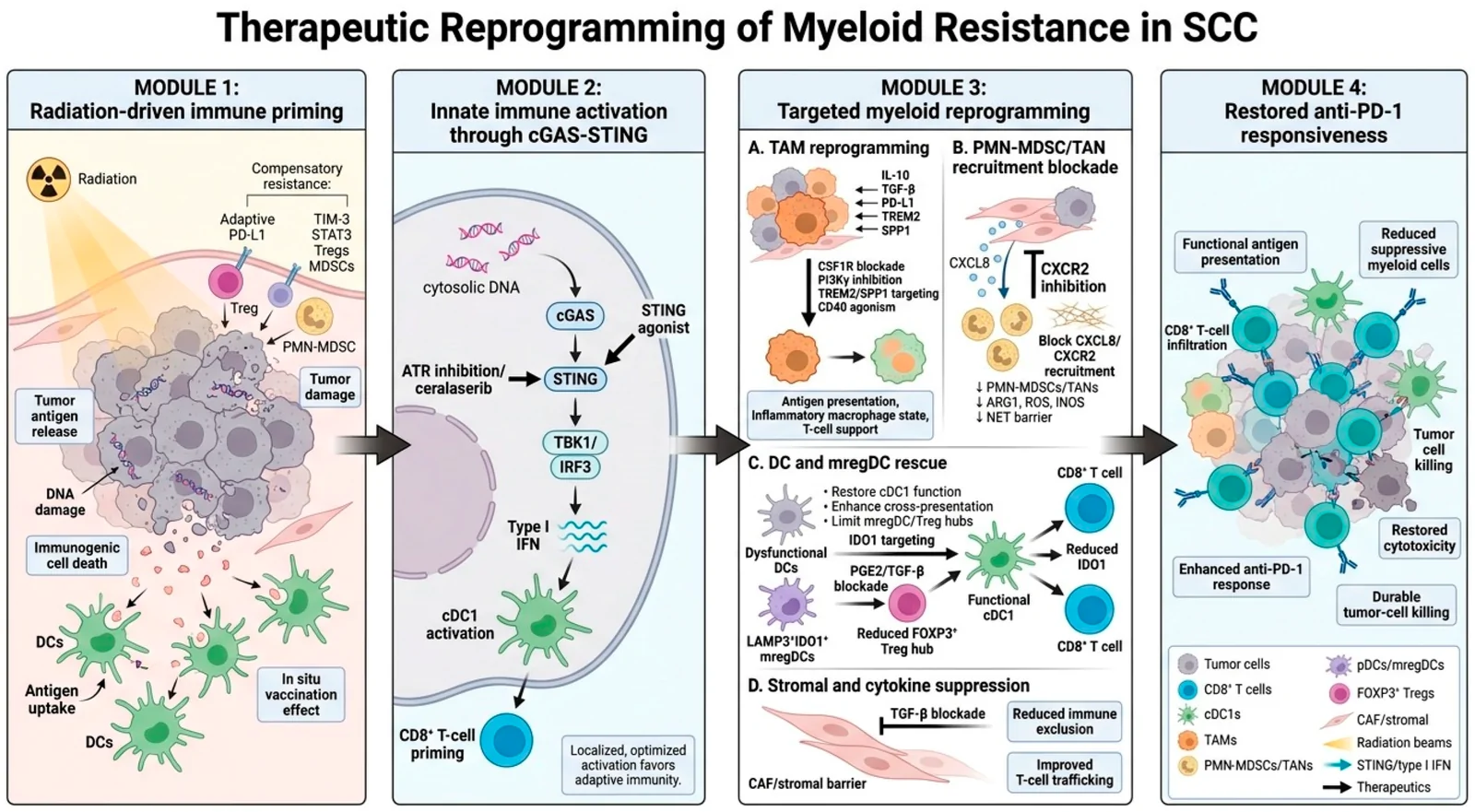
Therapeutic reprogramming of myeloid-mediated resistance to immune checkpoint blockade in squamous cell carcinoma. Radiation promotes immunogenic tumor cell death and antigen release. cGAS–STING activation (including STING agonists or ATR or Wee1 inhibition) enhances type I interferon signaling, cDC1 activation, and antigen cross-presentation. Concurrent targeting of suppressive myeloid populations, including TAMs (e.g., CSF1R, PI3Kγ, TREM2/SPP1, and CD40-directed therapies), PMN-MDSCs/TANs (CXCR2 inhibition), dysfunctional dendritic cells, and mregDC–Treg, restores antigen presentation, CD8^+^ T cell recruitment, and intratumoral cytotoxic activity. Arrows: down, downregulation or decreased infiltration.

Recognition of the central role of myeloid populations in shaping antitumor immunity has shifted therapeutic development beyond conventional approaches focused solely on tumor-cell eradication. Accumulating evidence indicates that suppressive myeloid populations are not merely passive biomarkers of immune dysfunction but active regulators of therapeutic response, making them attractive targets for intervention [20]. Consequently, contemporary therapeutic strategies increasingly seek to reprogram rather than deplete the immune TME, with the goal of overcoming myeloid-mediated immune suppression [107,108,109]. Among these approaches, radiation therapy and activation of innate immune sensing pathways have emerged as particularly promising strategies. Radiation can promote antigen release, DC activation, and T cell priming, functioning in some settings as an in situ vaccine [110,111,112,113,114,115], whereas activation of the cGAS-STING pathway stimulates type I interferon signaling, antigen cross-presentation, and tumor-specific T cell responses [116,117,118]. It is also important to note that radiation, STING agonism, and myeloid-targeted agents are not expected to act uniformly across myeloid subpopulations. Radiation preferentially depletes radiosensitive lymphoid populations while sparing tumor-associated macrophages, which are intrinsically radioresistant and can subsequently repolarize toward either an M1-like or M2-like phenotype depending on radiation dose and the post-radiation cytokine milieu [119]. STING agonism’s antitumor effect is similarly cell-type-restricted rather than acting broadly across the myeloid compartment: cDC1s specifically, rather than macrophages, monocyte-derived DCs, or granulocytic populations, have been shown to be required for STING agonist-mediated tumor rejection in preclinical models, and some STING agonist formulations (including ADU-S100) can paradoxically deplete rather than activate cDC1s within the injected tumor [120]. CXCR2 inhibition acts preferentially on PMN-MDSC/neutrophil recruitment via the ELR+ CXC chemokine axis, with comparatively little direct effect on monocytic MDSCs or macrophages, while CSF1R inhibition acts on monocyte/macrophage survival with minimal direct effect on the neutrophil compartment, consistent with the lineage-restricted receptor biology of CSF1R and CXCR2 discussed above [121]. Rational combination design should therefore match the intervention to the myeloid subpopulation dominating a given tumor’s suppressive landscape, rather than assuming any single agent broadly reprograms the entire myeloid compartment. Together, these therapeutic modalities illustrate how manipulation of innate immunity and myeloid-cell function may enhance responsiveness to immune checkpoint blockade in SCCs (Figure 2).

### 4.1. Radiation Therapy Reprograms the Myeloid Compartment

Radiation therapy has traditionally been viewed as a local cytotoxic modality; however, accumulating evidence indicates that radiation also functions as a potent regulator of antitumor immunity [122]. Beyond inducing tumor cell death, radiation promotes release of tumor-associated antigens, enhances DC cross-presentation, stimulates type I interferon signaling through innate immune sensing pathways, and facilitates recruitment of effector lymphocytes into the TME [122,123,124,125]. These observations led Demaria and colleagues to propose that radiation may function as an “in situ vaccine,” converting poorly immunogenic tumors into lesions capable of generating systemic antitumor immune responses [110,126]. Importantly, subsequent studies demonstrated that radiation-induced immune activation is not intrinsically sufficient for durable tumor control because compensatory immunosuppressive pathways frequently emerge following treatment.

Indeed, resistance to combined radiation and CTLA-4 blockade is associated with adaptive upregulation of PD-L1 and expansion of suppressive immune programs within the TME [127]. Addition of PD-L1 blockade restored therapeutic efficacy, suggesting that radiation simultaneously induces immune activation and compensatory resistance mechanisms [127]. These findings highlight that successful radioimmunotherapy requires not only generation of antitumor immunity but also interruption of suppressive pathways that limit its durability [110,127]. Subsequent work in HNSCC further reinforced the importance of combating these resistance programs. Resistance to combined radiotherapy and PD-L1 blockade was associated with increased TIM-3 expression on CD8+ T cells and Tregs, accompanied by accumulation of intratumoral Tregs [128]. Addition of TIM-3 blockade improved tumor control, enhanced cytotoxic T cell activity, and prolonged survival [128]. Similarly, STAT3 signaling has emerged as a major regulator of radiation-associated immunosuppressive remodeling [129]. Inhibition of STAT3 decreased infiltration of MDSCs, M2 macrophages, and Tregs, and concomitantly increased infiltration of M1 macrophages and effector T cells following radiation [129].

Emerging clinical studies further support that radiation can favorably reshape the TME when combined with immunotherapy. In the Neoadjuvant Immuno-Radiotherapy Trial, stereotactic body radiation therapy (SBRT) with nivolumab (anti-PD-1) prior to surgery in patients with locally advanced HNSCC showed major pathological response (mPR) and pathological complete response (pCR) rates of 86% and 67%, respectively, with clinical-to-pathological downstaging in 90% of treated patients [130]. Importantly, these response rates greatly exceeded those historically reported with neoadjuvant ICI alone, where mPR rates in HNSCC are generally limited to approximately 7–14% [130]. It should be noted that the Leidner et al. data, while striking, come from a single-arm phase 2 study; randomized trials directly testing radiation-ICI sequencing against ICI alone in SCC are still lacking. It is also important to recognize that radiation’s immunomodulatory effects are not uniformly beneficial: radiotherapy induces significant, often prolonged lymphopenia through direct cytotoxic effects on circulating lymphocytes transiting the irradiated field. In HNSCC specifically, 97% of patients develop lymphopenia during radiotherapy, and this is independently associated with worse survival [131]. Because lymphopenia risk scales with the volume of lymphoid-bearing tissue irradiated, radiation’s net immunological effect depends heavily on field size, dose, and fractionation, and can be immunosuppressive rather than immune-potentiating in a given patient, underscoring that radiation-immunotherapy combination outcomes are not uniformly favorable and are influenced by treatment technique [131]. Prior treatment history also likely shapes the myeloid compartment in ways that could inform combination strategy selection. Platinum-based chemotherapy can transiently deplete circulating myeloid populations while also inducing immunogenic cell death that promotes antigen release and dendritic cell activation, and radiation induces substantial and often prolonged lymphopenia, as discussed above [131], while also reshaping the local myeloid compartment through the mechanisms described in the radiation section. Patients entering ICI or myeloid-targeted combination therapy after platinum-based chemotherapy or radiation are therefore unlikely to have a treatment-naive myeloid landscape, yet none of the trials discussed in this review stratified myeloid-targeted therapy selection by prior treatment exposure, and this represents an unaddressed variable that could meaningfully affect which myeloid-targeted strategy, if any, is appropriate for a given patient. Similarly, mechanistic studies have also shown that radiation enhances DC recruitment and antigen presentation, promotes expansion of tumor-specific CD8+ T cells within tumor-draining lymph nodes, and facilitates trafficking of effector T cells into the TME [132]. Notably, omission of elective nodal irradiation in preclinical HNSCC models resulted in greater circulating antigen-specific CD4+ T cell responses and improved distant tumor control, highlighting the importance of preserving immune-priming niches during radioimmunotherapy [132,133,134]. Together, these findings suggest that radiation may function as a potent immune-modulating intervention capable of overcoming suppressive TME programs and enhancing responsiveness to ICI. The optimal sequencing of radiation and checkpoint blockade, however, remains unresolved: neoadjuvant trials to date have generally administered ICI concurrently with or shortly after radiation rather than systematically testing sequential alternatives, and head-to-head sequencing trials in SCC are lacking. A related question of timing applies to STING agonism, discussed next: preclinical data indicate that the dose and timing of STING activation critically determine whether the response supports adaptive antitumor priming or instead produces nonspecific inflammation [135].

### 4.2. STING Activation and Innate Immune Reprogramming

Innate immune activation through the cGAS-STING pathway has emerged as a rational strategy for overcoming tumors that fail to generate productive antitumor T cell responses. STING activation represents a method to initiate immunity from within the TME itself, particularly in tumors that lack spontaneous CD8+ T cell infiltration [116]. Cytosolic DNA sensing activates STING signaling [136,137], leading to TBK1/IRF3-dependent type I interferon production, DCDC activation, antigen cross-presentation, and subsequent priming of tumor-specific CD8+ T cells [116,138]. STING activation therefore provides a mechanistic bridge between innate immune sensing and adaptive T cell immunity. Further, the magnitude and localization of STING activation determine whether the response becomes durable and T cell mediated [135]. Intratumoral STING activation with the STING agonist ADU-S100 can produce tumor regression, but that high-dose tumor-ablative regimens and lower-dose immunogenic regimens are not equivalent [135]. Low-dose immunogenic activation generated local expansion of tumor-specific CD8+ effector T cells and supported durable antitumor immunity, whereas excessive activation produced systemic drug distribution and innate inflammatory effects that were less compatible with adaptive immune priming [135]. Importantly, the durable response required STING expression and interferon-alpha/beta receptor (IFNAR) signaling in hematopoietic cells, showing that the therapeutic effect was not merely due to direct tumor toxicity but dependent on type I interferon signaling [135].

Ataxia telangiectasia and Rad3-related protein (ATR) serve as central regulators of the cellular response to DNA damage and replication stress [139,140]. Pharmacologic inhibition of ATR with AZD6738 (ceralasertib) disrupts DNA repair pathways in cancer cells, leading to increased genomic instability and enhanced apoptotic cell death [139,140]. In addition to its direct antitumor activity, AZD6738 has been demonstrated to reshape the TME, promoting a more immunogenic state [139,140]. Recent work from our group further demonstrates that innate immune reprogramming can be achieved through modulation of the DNA damage and replication stress response in HNSCC [141]. In preclinical models, pharmacologic ATR inhibition with AZD6738 (ceralasertib) significantly enhanced the antitumor efficacy of PD-1 blockade [141]. This effect was associated with remodeling of the TME, including increased cytotoxic T cell activity, reduced immunosuppressive myeloid and Treg populations, and polarization toward a more inflammatory macrophage phenotype [141]. Mechanistically, these changes were linked to activation of cGAS-STING signaling and suppression of STAT3 activity, suggesting that ATR inhibition may augment immunotherapy responses through coordinated effects on both innate and adaptive immunity [141].

Clinical experience with direct STING agonism in HNSCC specifically comes primarily from the first-in-human trial of MK-1454 (ulevostinag), in which intratumoral MK-1454 combined with pembrolizumab produced a disease control rate of 48% and partial responses in 6 of 25 patients (3 of them HNSCC) across the combination-therapy arm, compared to a 20% disease control rate and no responses with MK-1454 monotherapy [142]. This informed a subsequent randomized phase 2 trial (NCT04220866) directly comparing intratumoral MK-1454 plus pembrolizumab against pembrolizumab alone in first-line recurrent/metastatic HNSCC, with early interim reporting suggesting a higher objective response rate for the combination (50%) than pembrolizumab alone (10%) in a small initial cohort. By contrast, the competing STING agonist ADU-S100 produced considerably lower response rates in its own first-in-human trial. Together, these data indicate that STING agonism has generated proof-of-concept clinical activity in HNSCC, but that this activity appears agent- and dose-dependent rather than a guaranteed class effect. It is important to temper the encouraging early clinical signal described above with the broader biology of STING signaling, which is not uniformly antitumor. Clinical development of STING agonists has been consistently hampered by poor systemic pharmacokinetics, necessitating intratumoral rather than systemic dosing, and by dose-limiting systemic inflammatory toxicity at doses sufficient for antitumor activity. More fundamentally, chronic (as opposed to acute) STING pathway activation—particularly in tumors with chromosomal instability—can paradoxically promote tumor progression: chromosomal instability-driven, sustained cGAS-STING signaling has been shown to drive a pro-metastatic tumor microenvironment through non-canonical NF-κB signaling and type I interferon tachyphylaxis, rather than durable antitumor immunity [143]. This dichotomy suggests that the timing, dose, and duration of STING activation—rather than activation per se—determine whether the net effect is antitumor or protumor, and that the acute, high-dose, intratumoral activation achieved in trials such as MK-1454 may not generalize to strategies aiming for more sustained STING pathway engagement. By restoring innate immune sensing and overcoming suppressive myeloid programs, these strategies may convert immunologically resistant SCCs into tumors capable of mounting effective responses to immune checkpoint blockade. Collectively, these studies identify STING signaling as a critical link between innate and adaptive immunity, promoting DC activation, type I interferon signaling, antigen presentation, and CD8+ T cell priming. Importantly, they demonstrate that therapeutic activation of this pathway can be achieved through multiple approaches, including direct STING agonism and indirect activation through ATR inhibition.

### 4.3. Emerging Myeloid-Targeted Therapeutic Strategies

Recognition of the central role of suppressive myeloid populations has prompted the development of therapeutic strategies aimed not only at depleting these cells but also at selectively reprogramming their function. Early macrophage-directed approaches focused on inhibition of the colony-stimulating factor-1 receptor (CSF1R), a key survival pathway for TAMs [144]. Early clinical studies with CSF1R-targeting agents, including pexidartinib [145] and monoclonal antibodies directed against CSF1R, demonstrated clear evidence of macrophage depletion and modulation within the TME [146]. However, clinical efficacy as monotherapy has generally been limited, and treatment has been associated with dose-limiting toxicities and adaptive resistance mechanisms that may restrict durable responses [147]. These observations have shifted attention toward combination strategies, particularly those integrating CSF1R inhibition with ICIs or other immunomodulatory therapies, with the goal of overcoming compensatory immunosuppressive pathways and enhancing antitumor immunity [147].

Clinically, CSF1R inhibition in SCC has largely been tested as part of early-phase combination trials rather than as a registration study; for example, the CSF1R inhibitor pexidartinib has been combined with pembrolizumab in a phase 1/2a basket trial enrolling solid tumor cohorts [148], and broader analyses of CSF1R inhibitor trials across solid tumors report that pharmacodynamic evidence of macrophage depletion is consistently observed, but objective response rates as monotherapy or in combination with checkpoint blockade have remained modest outside of tenosynovial giant cell tumor, a non-malignant, CSF1R-driven neoplasm in which CSF1R inhibitors are highly active [43]. CSF1R inhibition has also been shown, in preclinical glioma models, to trigger compensatory upregulation of alternative pro-tumor survival signals despite continued target blockade: acquired resistance to CSF1R inhibition (BLZ945) was found to be driven by macrophage-derived IGF-1 activating IGF-1R/PI3K signaling in tumor cells, allowing tumor regrowth even with sustained CSF1R pathway suppression, and combined blockade of PI3K and IGF-1 signaling alongside CSF1R inhibition substantially improved outcomes in this model [149]. A related mechanism involving compensatory CSF2 (GM-CSF)-driven macrophage activation has also been described as a route to CSF1R-inhibitor resistance. This compensatory potential is consistent with the modest single-agent clinical activity of CSF1R inhibitors discussed above and suggests that durable myeloid reprogramming may require simultaneously targeting compensatory pathways rather than a single node, though these specific compensatory pathways have been characterized in glioma models and have not yet been examined in SCC specifically. This distinction between pharmacodynamic activity and clinical benefit is a recurring theme across the myeloid-targeted agents discussed in this section and is addressed further at the end of this section.

More recent work suggests that these limitations may arise, in part, from the biological heterogeneity of TAMs. As discussed in the previous section, single-cell and spatial profiling studies have identified distinct macrophage states across solid tumors, including recurrent TREM2+ [150,151,152], SPP1+ [153,154], and CXCL9+ populations [154,155,156,157]. Several of these classifications were originally derived from pan-cancer single-cell atlases spanning multiple tumor types rather than SCC-specific cohorts, and readers should note that the strength of evidence for each population’s presence and function varies accordingly between subtypes in which it has been directly profiled and subtypes where its relevance is inferred by extension. Hence, broad macrophage depletion approaches may simultaneously eliminate both immunosuppressive and immunostimulatory macrophage subsets [107]. Consequently, current efforts increasingly emphasize selective reprogramming or targeting of specific TAM populations rather than indiscriminate macrophage depletion [107].

A critical implication of this plasticity is that myeloid states are not fixed cell-autonomous identities but dynamic responses to microenvironmental cues, meaning that a given TAM or MDSC population can transition between suppressive and stimulatory phenotypes depending on local metabolic, cytokine, and spatial context. This has direct therapeutic relevance: because depletion strategies remove myeloid cells regardless of their functional state, they eliminate both immunosuppressive and antitumor myeloid populations indiscriminately, which likely explains why broad myeloid depletion has generally underperformed relative to therapies that instead redirect existing myeloid cells toward a more immunostimulatory state. Understanding the specific signals that drive transitions between these states, rather than simply cataloguing the states themselves, is therefore a prerequisite for designing reprogramming strategies with predictable clinical effects. An open question is whether selective depletion of a well-defined pathogenic subset—for example, CSF1R-dependent TAMs specifically, as opposed to broad macrophage depletion—might be more feasible than reprogramming strategies that require precisely tuning a cell’s functional state rather than simply removing it. Selective depletion has the practical advantage of a more tractable pharmacologic endpoint (target engagement and cell number reduction are directly measurable), whereas reprogramming requires evidence that a cell’s transcriptional and functional state has shifted in the intended direction, which is harder to establish and monitor clinically. However, as discussed above, CSF1R-dependent TAMs are not a functionally homogeneous population, so selective depletion restricted to this subset may still face the same indiscriminate-elimination problem at a smaller scale unless depletion can be further restricted to specifically pathogenic sub-states. An additional layer of myeloid regulation not yet discussed is tumor immunometabolism. Hypoxic tumor regions and tumor-derived lactate reshape myeloid polarization directly: hypoxia stabilizes HIF-1α in myeloid cells, and tumor-derived lactate accumulation and the resulting extracellular acidification together drive myeloid cells toward a pro-tumoral, immunosuppressive phenotype characterized by expression of IL-6, PD-L1, and VEGF [158]. This same metabolically hostile, lactate-rich, hypoxic environment is well established to restrict the substrates available to infiltrating T cells more broadly, independent of any direct suppressive signaling; because myeloid cells are both abundant and metabolically active within this environment, their glycolytic and lactate-handling programs likely compound this substrate restriction, though whether myeloid cells specifically outcompete T cells for glucose, as opposed to tumor cells doing so, has not been directly established and remains an open question. This immunometabolic dimension likely interacts with, rather than operates independently of, the cytokine- and checkpoint-mediated suppression mechanisms discussed throughout this review, and represents an underexplored axis for combination therapy design in SCC specifically. Tertiary lymphoid structures (TLS)—ectopic lymphoid aggregates containing organized T-cell and B-cell zones—have emerged as a favorable prognostic and predictive feature in ICI-treated SCC, and their formation and function are myeloid-dependent: mature dendritic cells and specific macrophage subsets within and adjacent to TLS support local antigen presentation and T-cell priming, and a TLS-associated gene signature enriched for T follicular helper cell activity has been linked to improved survival in HNSCC, particularly in HPV-positive disease. TLS prevalence itself differs substantially across SCC subtypes, having been reported in over 95% of LUSC tumors compared with approximately 21% of HNSCC tumors, a difference that may partly explain the differential ICI efficacy across these subtypes discussed elsewhere in this review. The myeloid populations that organize and maintain TLS represent a functionally distinct, immunostimulatory counterpart to the suppressive myeloid programs emphasized throughout this review, and their preservation—rather than depletion—should be an explicit design consideration for myeloid-targeted combination strategies going forward [159]. Beyond direct cytokine- and metabolite-mediated myeloid reprogramming discussed above, tumor-derived extracellular vesicles (exosomes) represent an additional mechanism by which tumors educate myeloid cells, including at distant, pre-metastatic sites. Tumor exosomes are taken up preferentially by myeloid cells and can polarize macrophages toward an immunosuppressive, PD-L1-high phenotype through Toll-like receptor and NF-κB-dependent signaling, and can also promote differentiation of myeloid progenitors toward an MDSC phenotype at sites distant from the primary tumor, contributing to pre-metastatic niche formation [160]. Because exosomal cargo is tumor-derived and detectable in peripheral blood, tumor exosomes represent both a potential therapeutic target—for example, through inhibition of exosome biogenesis or uptake—and a potential liquid biopsy biomarker of myeloid reprogramming that would not require the serial tissue biopsies whose feasibility is discussed elsewhere in this review. This mechanism has not been specifically characterized in SCC and represents an unexplored area relevant to the myeloid-targeted strategies discussed throughout.

Although the mechanistic evidence reviewed above is drawn predominantly from HNSCC, independent single-cell profiling of cSCC and LUSC reveals both convergent and site-specific myeloid biology. SPP1+ TAMs, the dominant immunosuppressive macrophage state emphasized throughout this review in HNSCC, also emerge as a defining macrophage population in both cSCC and LUSC: in cSCC, recurrent tumors show a T-cell-excluded, SPP1+ TAM-enriched microenvironment relative to primary tumors, with two functionally distinct SPP1+ subsets characterized by high phagocytic versus high angiogenic activity [161]; in LUSC, single-cell comparison against lung adenocarcinoma found that SPP1+ macrophages are the dominant tumor-associated macrophage subtype in LUSC specifically, in contrast to a FABP4+ dominant subtype in adenocarcinoma [162]. This convergence suggests that SPP1+ TAM programs may represent a genuinely shared, anatomically independent node of myeloid-mediated immunosuppression across SCC subtypes, strengthening the rationale for the reprogramming strategies discussed in this review. At the same time, site-specific differences are apparent: the cSCC data implicate this phenotype specifically in tumor recurrence rather than treatment-naive disease, a temporal association not yet described in HNSCC, while the LUSC data show that squamous histology itself, independent of anatomic site, is associated with a distinct dominant macrophage identity compared to adenocarcinoma arising in the same organ, implying that tissue-of-origin, local antigenic exposure (UV radiation in skin, tobacco carcinogens and HPV status in the aerodigestive tract), and organ-specific stromal architecture likely shape myeloid programming in ways that are not yet mapped in a single unified dataset. These site-specific differences likely reflect distinct upstream etiological signals rather than a single convergent pathway: HPV-driven HNSCC exposes myeloid cells to viral antigens and a chronic type I interferon-skewed inflammatory milieu, whereas UV-driven cSCC exposes the myeloid compartment to a high burden of UV-induced neoantigens and photodamage-associated danger signals without a viral component, and tobacco/alcohol-driven HPV-negative HNSCC and LUSC instead reflect chronic chemical carcinogen exposure. Because these upstream signals differ mechanistically, myeloid polarization programs converging on a shared phenotype (e.g., SPP1+ TAM dominance) across SCC subtypes should be interpreted as convergent functional outcomes arising from distinct etiological inputs, rather than evidence of one shared causal pathway. We therefore distinguish, and encourage readers to distinguish, between a shared myeloid mechanism (identical molecular pathways operating across subtypes) and a convergent myeloid phenotype (different upstream signals independently producing similar functional myeloid states, such as SPP1+ TAM dominance or PMN-MDSC-mediated T-cell exclusion). The evidence summarized in this review is most consistent with the latter: convergence at the level of functional myeloid output, not necessarily identity of the driving molecular program. These findings support the shared-mechanism framework proposed in this review while also indicating that site-specific validation, rather than direct extrapolation from HNSCC, will be necessary before myeloid-directed therapeutic strategies are generalized across SCC subtypes. With this caveat in mind, the reprogramming strategies discussed below have so far been evaluated almost exclusively in HNSCC, and their preclinical and early clinical signals should be read as hypothesis-generating for other SCC subtypes rather than directly transferable. Directly testing this generalizability would require a harmonized, cross-anatomic-site single-cell and spatial transcriptomic atlas of HNSCC, cSCC, ESCC, and LUSC processed through a single analytical pipeline with matched staining panels and cell-calling thresholds—an approach that would resolve the platform- and methodology-driven heterogeneity that currently prevents direct comparison of the individually published site-specific datasets discussed above. A systematic comparative meta-analysis restricted to studies using compatible single-cell platforms and myeloid marker panels would represent a more immediately achievable, lower-cost interim step toward the same goal

Beyond macrophage depletion, PI3Kγ inhibition is another promising approach of functional myeloid reprogramming. Macrophage PI3Kγ acts as a molecular switch controlling immune suppression: PI3Kγ activation promotes an immunosuppressive transcriptional program, whereas genetic or pharmacologic PI3Kγ inhibition shifts macrophages toward an immune-stimulatory state, increases CD8+ T cell activation, and enhances responsiveness to ICI [163]. Consistent with these findings, emerging clinical studies with the PI3Kγ inhibitor eganelisib have demonstrated pharmacodynamic evidence of myeloid reprogramming [164] and encouraging activity in combination with ICI, supporting the feasibility of therapeutically redirecting suppressive macrophage states rather than eliminating them [107]. It is also important to note that PI3Kγ is expressed across multiple myeloid lineages and its inhibition does not act uniformly on macrophages and neutrophils: PI3Kγ blockade shifts macrophage polarization toward an immunostimulatory state as described above, but its effects on neutrophil chemotaxis and PMN-MDSC function are less well characterized and may differ mechanistically. Because the relative abundance of macrophages versus neutrophil-lineage cells varies substantially between tumors and SCC subtypes, PI3Kγ inhibitor efficacy is likely context-dependent on this underlying myeloid composition, and trials that do not stratify by baseline macrophage-to-neutrophil ratio may underestimate the agent’s efficacy in the subset of tumors where it is mechanistically best matched [163]. SCC-specific clinical data for eganelisib remain preliminary and derive mainly from a single early-phase dataset: in the SCCHN expansion cohort of the phase 1/1b MARIO-1 trial, eganelisib plus nivolumab produced one complete response and stable disease in 36% of evaluable patients, with a formal phase 2 study (MARIO-8) subsequently initiated to evaluate eganelisib plus pembrolizumab in first-line recurrent/metastatic HNSCC [44]. As of this writing, efficacy results from MARIO-8 have not yet been reported in the peer-reviewed literature, underscoring that translation of PI3Kγ inhibition into an approved SCC therapy is not yet established. Beyond PI3Kγ-selective inhibition, broader PI3K/mTOR blockade with gedatolisib has also been shown to reshape the immunosuppressive TME in HNSCC, reducing regulatory T cell infiltration and tumor hypoxia while resensitizing cisplatin-resistant, Nrf2-hyperactivated tumors to chemotherapy [165].

Targeting myeloid recruitment pathways represents another promising therapeutic strategy. The CXCL-CXCR2 signaling axis plays a central role in the recruitment of suppressive myeloid populations to the TME and has been implicated in immune evasion across multiple SCC subtypes, including HNSCC [166,167]. Preclinical studies have demonstrated that CXCR2 inhibition can reduce the accumulation of immunosuppressive myeloid cells, enhance T cell infiltration, and improve responses to ICI [166]. These findings support the broader concept that disrupting myeloid-cell recruitment may convert immune-excluded tumors into more immunologically responsive lesions and provide a rationale for combining CXCR2-targeted therapies with ICIs in SCC. Direct clinical evidence in SCC is limited to early-phase combination data: in the dose-expansion phase of the SCORES trial, the CXCR2 antagonist AZD5069 combined with durvalumab produced an objective response rate of 5% among 22 PD-L1-inhibitor-naive and 10% among 20 PD-L1-inhibitor-pretreated patients with recurrent/metastatic HNSCC, and the addition of AZD5069 did not improve response rate over durvalumab alone while adding toxicity, with treatment-related adverse events occurring in 76% of patients [168]. These modest results, despite a strong preclinical rationale, illustrate that CXCR2 inhibition in SCC has not yet been validated as a clinically transformative strategy. It is important to view these encouraging preclinical and early-phase signals against a broader record of negative or inconclusive myeloid-targeted trials. STING agonism illustrates this point: the competing agent ADU-S100 produced partial responses in fewer than 10% of patients across tumor types in its first-in-human trial, and its combination with pembrolizumab in first-line recurrent/metastatic HNSCC did not proceed to the efficacy data stage reported for MK-1454/ulevostinag [142]. Similarly, CSF1R inhibitors have shown consistent target engagement across cancers but have not demonstrated meaningful single-agent activity in solid tumors outside tenosynovial giant cell tumor [43], and CXCR2 inhibition added toxicity without improving response rate in the one HNSCC trial reported to date [168]. The limited single-agent activity of CSF1R, PI3Kγ, and CXCR2 inhibitors documented above raises a specific concern for their use as ICI combination partners: an agent with minimal monotherapy activity may still lack sufficient target engagement or pathway modulation to meaningfully alter the tumor immune contexture even when combined with ICI, and combination trial designs that do not include a mechanistic biomarker confirming on-target myeloid reprogramming cannot distinguish a true combination benefit from an ICI effect alone. Other than the small-molecule and antibody approaches discussed above, engineered cellular approaches to myeloid reprogramming have recently entered early clinical testing. Chimeric antigen receptor macrophages (CAR-Ms) are engineered to phagocytose tumor cells, activate the local TME, and enhance antigen presentation to T cells. In a first-in-human phase 1 trial of CT-0508, an anti-HER2 CAR-M, in HER2-overexpressing solid tumors, treatment was feasible to manufacture and well tolerated with no dose-limiting toxicities, and correlative analysis of serial biopsies confirmed CAR-M trafficking to tumors with subsequent TME remodeling and expansion of CD8+ T cells, with stable disease as the best response in 44% of HER2 3+ tumors [169]. Bispecific antibodies engaging both a myeloid checkpoint (e.g., CD47/SIRPα) and a tumor antigen represent a related emerging strategy aiming to combine targeted phagocytosis with myeloid reprogramming. Neither approach has yet been tested in SCC specifically, but the CAR-M feasibility and safety data support their consideration as future combination partners for ICI in this disease group. Beyond CSF1R, PI3Kγ, and CXCR2 inhibition, several additional myeloid- and innate-immune-directed strategies are under early clinical investigation in SCC. CD40 agonism, which licenses dendritic cells and can reprogram TAMs toward an antigen-presenting phenotype, has shown proof-of-concept activity in combination with anti-PD-1 in melanoma, where sotigalimab plus nivolumab produced an objective response rate of 15% in patients with confirmed progression on prior PD-1 blockade [170]; a dedicated phase 1 trial combining a CD40 agonist with PD-1 blockade in resectable HPV-negative HNSCC is ongoing (NCT06159621), though SCC-specific efficacy data are not yet available. TLR agonism has in fact been tested more extensively in HNSCC than the other targets discussed here: the TLR8 agonist motolimod was evaluated in the randomized, placebo-controlled phase 2 ACTIVE8 trial in combination with the EXTREME regimen in recurrent/metastatic HNSCC, but did not improve progression-free or overall survival in the intent-to-treat population, although a preplanned subgroup analysis suggested benefit in HPV-positive patients, a finding that requires prospective confirmation [171]. CCR2 inhibition, which blocks monocyte/TAM recruitment via the CCL2–CCR2 axis, remains earlier in development still, with preclinical and early-phase data concentrated in other tumor types and no completed clinical trial identified specifically in SCC to date. Taken together with the CSF1R, PI3Kγ, CXCR2, and STING data discussed throughout this section, these findings underscore that essentially all myeloid- and innate-immune-targeted strategies discussed in this review; CD40 agonists, PI3Kγ inhibitors, STING agonists, TLR agonists, CXCR2 inhibitors, and CCR2 inhibitors alike; remain in early-phase clinical development or earlier, and that current evidence is insufficient to determine which, if any, will translate into a clinically meaningful addition to ICI therapy in SCC. These outcomes do not invalidate the myeloid-reprogramming rationale, but they do indicate that pharmacodynamic evidence of myeloid modulation has repeatedly failed to translate into clinical benefit when tested as monotherapy or in unselected populations, reinforcing that rational, biomarker-guided combination strategies, rather than myeloid-targeting alone, are likely required.

Taken together, the translational limitations of myeloid-targeted therapy in SCC are best understood as four distinct but related problems rather than a single generic caveat: modest single-agent efficacy outside of narrow disease contexts (e.g., CSF1R inhibitors in tenosynovial giant cell tumor); resistance mechanisms that remain poorly characterized at the level of individual myeloid subsets; toxicity signals, including CSF1R-associated hepatotoxicity and PI3Kγ-associated cytopenias, that have not been systematically studied in combination with checkpoint blockade; and the absence of any prospectively validated biomarker capable of identifying which patients are likely to benefit from a given myeloid-targeted approach. None of these four problems is solved by the encouraging preclinical rationale summarized in Figure 2, and each will require dedicated clinical and correlative work before myeloid-targeted therapy can be considered translationally mature.

Additional strategies seek to restore productive communication between innate and adaptive immunity. Beatty and colleagues showed that CD40 agonism altered the pancreatic tumor stroma and produced antitumor effects in both mouse models and patients, with macrophage activation contributing to stromal remodeling and tumor regression rather than simple T cell-dependent killing [172]. In parallel, STING agonism represents another approach to innate immune activation. Optimized intratumoral STING activation with ADU-S100 generated tumor-specific CD8+ T cell expansion, durable antitumor immunity, and improved efficacy of ICI, but only when the magnitude and localization of STING activation supported adaptive immune priming rather than nonspecific inflammation [135].Collectively, these therapeutic strategies demonstrate that the myeloid compartment is not merely a biomarker of immune resistance but an actionable determinant of treatment response. CSF1R blockade, PI3Kγ inhibition, CXCR2 inhibition, and STING activation each target different aspects of the same biological problem: suppressive myeloid survival, recruitment, polarization, or failure of innate-to-adaptive immune communication. These findings directly support the central argument of this review that improving immunotherapy outcomes across SCCs will likely require strategies that reprogram the myeloid TME in parallel with ICI. It bears emphasizing that essentially all combination data discussed above derive from single-arm or small randomized phase 1/2 studies; no myeloid-targeted agent has yet been tested against ICI alone in an adequately powered phase 3 trial in SCC, so claims of added benefit over ICI monotherapy remain hypothesis-generating rather than established.

## 5. Conclusions

Although ICI have transformed the treatment landscape of SCC, durable responses remain limited in a substantial proportion of patients. Accumulating evidence suggests that suppressive myeloid programs represent a convergent functional outcome underlying both primary and acquired resistance to immunotherapy across SCC subtypes. TAMs, MDSCs, TANs, and dysfunctional DCs collectively shape the balance between immune activation and immune suppression, thereby influencing therapeutic responsiveness. Importantly, emerging strategies capable of reprogramming these myeloid populations; including radiation therapy, STING activation, and targeted myeloid-directed interventions; have demonstrated encouraging pre-clinical and early clinical activity. Continued integration of spatial biology, systems immunology, and translational therapeutics will be essential for defining actionable myeloid vulnerabilities and ultimately improving outcomes for patients with SCC.

## 6. Future Directions

Several priorities emerge from the evidence reviewed here. First, most mechanistic insight into myeloid-mediated resistance has been generated in HNSCC and preclinical models, comparable spatial and single-cell characterization of the myeloid compartment has only recently begun to extend beyond HNSCC to LUSC and cSCC individually (see below), and cross-subtype profiling using harmonized methodology will still be needed to determine whether the TAM, TAN, and dendritic cell states described here represent a shared program of resistance or are subtype-specific. One dedicated comparative synthesis has attempted to bridge this gap across the aerodigestive tract SCC subtypes: van Duijvenvoorde et al. compiled flow cytometric data showing that myeloid lineage cells comprise approximately 50% of CD45+ tumor-infiltrating immune cells in LUSC specifically, with neutrophilic granulocytes accounting for 20% and monocytes 10% of this population (60% of the latter displaying an HLA-DRlow, monocytic-MDSC phenotype); however, the corresponding HNSCC and ESCC literature they reviewed reported myeloid infiltration only qualitatively, precluding a harmonized quantitative comparison even across these three sites, and cutaneous SCC was not addressed at all. Notably, that same synthesis characterized MDSC- and TAM-mediated immunosuppression as a feature shared across HNSCC, ESCC, and LUSC rather than a subtype-differentiating one, consistent with the shared-mechanism framework proposed in this review [159]. Second, functional TAM states such as TREM2+, SPP1+, and CXCL9+ populations were largely defined using single-cell transcriptomic clustering; validating these states with spatially resolved platforms and linking them prospectively to ICI outcomes will be necessary before they can be used as predictive biomarkers rather than descriptive correlates. This distinction matters clinically: a prognostic biomarker identifies patients with worse outcomes regardless of treatment received, while a predictive biomarker specifically identifies patients who benefit differentially from a given therapy. Of the biomarkers discussed in this review—including CD163, CSF1R expression, and CXCR2 pathway activity—essentially all have been only validated as prognostic markers, correlating with survival independent of treatment, rather than as predictive markers demonstrated in a randomized, biomarker-stratified trial to identify which patients specifically benefit from myeloid-targeted combination therapy. Using a prognostic marker as though it were predictive risks selecting patients who have worse outcomes generally, rather than patients who are more likely to benefit from the specific intervention being tested, and this distinction has not been rigorously tested for any biomarker discussed in this review. To move from acknowledging this gap toward a concrete path forward, we propose that candidate predictive biomarkers should be prioritized based on their mechanistic alignment with the specific agent under consideration: for CSF1R inhibitors, baseline intratumoral CSF1R+ TAM density by multiplex immunofluorescence or spatial transcriptomics; for CXCR2 inhibitors, baseline PMN-MDSC/TAN density and CXCL8 pathway activity; for PI3Kγ inhibitors, baseline macrophage PI3Kγ pathway activation signatures; and for STING agonists, baseline cGAS-STING pathway activity and chromosomal instability status, given the dichotomous acute-versus-chronic activation biology discussed above. Each of these candidates would require prospective validation in a biomarker-stratified randomized trial, rather than the correlative, non-randomized biomarker analyses that constitute most current evidence, before being considered predictive rather than merely associative. Third, because myeloid-targeted agents (CSF1R, PI3Kγ, and CXCR2 inhibitors) have shown limited efficacy as monotherapy, future trials should prioritize rational combinations with ICI, with correlative spatial and single-cell endpoints built in to determine which myeloid states are being reprogrammed rather than depleted. Relatedly, most existing reprogramming studies report shifts in myeloid composition or polarization markers as a correlative or pharmacodynamic endpoint rather than formally testing whether the magnitude of that shift predicts clinical response; without paired pre/post-treatment sampling powered for this correlation, it remains unclear whether myeloid biomarker changes are causally linked to outcome or simply co-occur with it. Also, integrating myeloid-TME profiling with clinical and exposure variables, such as smoking status, HPV status, and treatment setting, may help explain the variability in ICI response within a single SCC subtype and not only between subtypes, and could inform patient stratification for myeloid-directed combination therapy. A further limitation underlying much of the mechanistic evidence summarized in this review is the reliance on preclinical models with known constraints for studying human myeloid biology. Syngeneic mouse models retain an intact immune system but exhibit substantial species-specific differences from human myeloid cells, including differences in TLR expression, macrophage polarization marker repertoires, and MDSC surface phenotypes, such that a mouse-defined ‘TAM’ or ‘MDSC’ subset does not necessarily correspond one-to-one with its human counterpart. Patient-derived xenografts better preserve human tumor cell biology but are typically established in immunodeficient mice lacking a functional adaptive immune system and, in most standard models, lacking human myeloid cells altogether, limiting their utility for myeloid-specific mechanistic study without humanization. Humanized mouse models partially address this gap but introduce their own limitations, including incomplete human myeloid reconstitution and graft-versus-host effects. These model limitations mean that mechanistic claims regarding myeloid reprogramming strategies discussed throughout this review—particularly those supported primarily by murine data—should be considered provisional pending confirmation in human tissue or clinical correlative studies.

Finally, any combination strategy layering a myeloid-targeted agent onto standard ICI carries added drug acquisition cost, monitoring burden, and site-of-care complexity that will need to be weighed against incremental benefit as these regimens mature toward practice. This cost consideration is not merely a secondary implementation detail: SCC arising from tobacco, alcohol, and UV-associated etiologies disproportionately affects populations in low- and middle-income settings and in under-resourced healthcare systems even within high-income countries, and a combination strategy requiring two or more high-cost biologic or targeted agents alongside ICI may be economically inaccessible to the majority of the global SCC patient population regardless of its biological efficacy. Future trials of myeloid-targeted combination strategies should incorporate cost-effectiveness analysis and consider dosing or sequencing strategies designed to minimize total combination-agent exposure, rather than assuming efficacy alone determines clinical adoption.

An important unresolved question is whether myeloid-mediated resistance reflects a pre-existing tumor state present before ICI exposure, or an adaptive response that emerges during treatment as tumors are selected for or induced toward an immunosuppressive myeloid phenotype. This distinction has direct therapeutic implications: pre-existing myeloid-high tumors may require upfront combination therapy from treatment initiation, whereas treatment-emergent myeloid reprogramming might be addressed reactively, guided by on-treatment biopsy or liquid biomarker changes, without requiring combination therapy in patients who respond adequately to ICI monotherapy. Almost none of the trials discussed in this review incorporated serial, on-treatment myeloid profiling capable of distinguishing these scenarios, and this represents a significant gap in the field’s ability to rationally sequence myeloid-targeted therapy relative to ICI initiation. An additional source of inter-patient heterogeneity not addressed above is the gut microbiome, which has been shown to shape both myeloid cell polarization and ICI response. A practical barrier to implementing any of the biomarker-guided or temporally informed strategies discussed above is tissue accessibility: serial biopsies capable of tracking myeloid dynamics during therapy are frequently not feasible in SCC, given tumor location (e.g., laryngeal or hypopharyngeal HNSCC, esophageal ESCC), procedural risk, and patient tolerability of repeated invasive sampling. This constraint favors development of minimally invasive alternatives—including the circulating tumor exosome and cell-free biomarkers discussed above, peripheral blood myeloid immunophenotyping, and imaging-based approaches—over strategies that presuppose the feasibility of serial tissue sampling, and future correlative studies in SCC should prioritize validating such minimally invasive approaches against tissue-based ground truth where biopsy is feasible, rather than assuming serial biopsy access will be broadly available in clinical practice.

Commensal gut bacteria prime tumor-infiltrating myeloid cells via Toll-like receptor signaling, and specific bacterial taxa are differentially enriched in ICI responders versus non-responders, with taxa such as Bacteroidales positively associated with both regulatory T-cell and MDSC frequency in some cohorts [173]. Because none of the SCC trials discussed in this review incorporated microbiome profiling, the extent to which microbiome-driven myeloid polarization contributes to the inter-patient variability in ICI response described throughout this manuscript remains unknown and represents an unexplored source of heterogeneity specifically within SCC.

## Figures and Tables

**Table 1:** Landmark SCC immunotherapy trials.

SCC Type	Trial	Phase	Setting	Regimen	Comparator	Endpoint	Key Result	Significance	Ref
HNSCC	CheckMate 141	III	Platinum-refractory recurrent/metastatic	Nivolumab 3 mg/kg every 2 wk	Investigator’s choice chemotherapy	OS; PFS	Median OS 7.5 vs. 5.1 mo: HR 0.70 (97.73% CI, 0.51 to 0.96); *p* = 0.01, Median PFS 2.0 vs. 2.3 mo; HR 0.89 (95% CI, 0.70–1.13); *p* = 0.32	First PD-1 inhibitor to improve survival in R/M HNSCC	[28]
HNSCC	KEYNOTE-048	III	First-line recurrent/metastatic	Pembrolizumab ± chemotherapy: pembrolizumab 200 mg IV every 3 wk; platinum backbone with carboplatin AUC 5 or cisplatin 100 mg/m^2^ every 3 wk for up to 6 cycles; 5-FU 1000 mg/m^2^/day every 3 wk	EXTREME regimen: cetuximab loading dose 400 mg/m^2^ IV day 1, then 250 mg/m^2^ IV wk	OS	Pembrolizumab alone vs. cetuximab + chemotherapy In CPS ≥ 20, median OS 14.9 vs. 10.7 mo; HR 0.61 (95% CI, 0.45–0.83); *p* = 0.0007 In CPS ≥ 1, median OS 12.3 vs. 10.3 mo; HR 0.78 (95% CI, 0.64–0.96); *p* = 0.0086. Pembrolizumab + chemotherapy vs. cetuximab + chemotherapy In CPS ≥ 20, median OS 14.7 vs. 11.0 mo; HR 0.60 (95% CI, 0.45–0.82); *p* = 0.0004 In CPS ≥ 1, median OS 13.6 vs. 10.4 mo; HR 0.65 (95% CI, 0.53–0.80); *p* < 0.0001.	Established PD-1-based first-line standard	[8]
HNSCC	KEYNOTE-040	III	Previously treated recurrent/metastatic	Pembrolizumab 200 mg IV every 3 wk	Standard therapy	OS	HR 0.80 (95% CI, 0.65–0.98); OS 8.4 vs. 6.9 mo; *p*= 0.0161	Confirmed activity of PD-1 blockade in recurrent/metastatic disease	[29]
HNSCC	KEYNOTE-689	III	Resectable locally advanced	Perioperative pembrolizumab + SOC: 2 cycles neoadjuvant pembrolizumab; adjuvant risk-adjusted SOC, RT ± cisplatin, plus pembrolizumab every 3 wk for 45 wk	SOC alone: surgery followed by postoperative risk-adjusted SOC, RT ± cisplatin	EFS	In CPS ≥ 10, 36-mo EFS 59.8% vs. 45.9%; HR 0.66 (95% CI, 0.49–0.88); *p* = 0.004 In CPS ≥ 1, 36-mo EFS: 58.2% vs. 44.9%; HR 0.70 (95% CI, 0.55–0.89); *p* = 0.003	First positive perioperative immunotherapy trial in HNSCC	[9]
HNSCC	NIVOPOSTOP	III	Postoperative locally advanced	Nivolumab + adjuvant CRT: nivolumab 360 mg every 3 wk, then 480 mg every 6 wk for 6 cycles	Adjuvant CRT: radiation up to 66 Gy + cisplatin 100 mg/m^2^ every 3 wk for 3 cycles	DFS	3-yr DFS 63.1% vs. 52.5%, HR 0.76, (95% CI 0.60–0.98); *p* = 0.034	Supports incorporation of immunotherapy into postoperative management	[30]
cSCC	EMPOWER-CSCC-1	I/II	Advanced/metastatic	Cemiplimab 3 mg/kg IV every 2 wk; later dosing 350 mg every 3 wk	Single arm	ORR	95% CI 40–54.4%ORR ~47%; DOR 88.3%; updated ORR 44.8%; DOR not reached, *p* = NA	Led to first FDA approval of immunotherapy for cSCC	[31]
cSCC	KEYNOTE-629	II	Advanced/metastatic	Pembrolizumab 200 mg every 3 wk	Single arm	ORR, DCR	ORR 34.3%; 95% CI (25.3–44.2%) DCR 52.4%; 95% CI (42.4–62.2%)	Expanded role of PD-1 blockade in cSCC	[7]
cSCC	MATISSE	II	Resectable disease	Neoadjuvant nivolumab ± ipilimumab 3 mg/kg IV Wk 0 and 2 (2 doses total), Nivo + IPI: Nivolumab 3 mg/kg IV Wk 0 and 2 Ipilimumab IV Given in Wk 0 only (1 doses total)	Single arm	pRR, MPR	Nivolumab monotherapy: 55% pRR (45% MPR, 10% PPR); 95% CI, 31.5–76.9% Nivolumab + low-dose ipilimumab: 80% pRR (50% MPR, 30% PPR); 95% CI, 56.3–94.3% Between-group comparison: HR = NA; *p* value not reported MPR: 45% vs. 50%	Demonstrated feasibility of neoadjuvant immunotherapy	[32]
cSCC	C-POST	III	High-risk resected	Adjuvant cemiplimab 350 mg IV every 3 wk for 12 wk, followed by 700 mg IV every 6 wk for up to 36 wk; 48 wk total	Placebo	DFS	24-mo DFS 87.1% vs. 64.1%; HR 0.32 (95% CI, 0.20–0.51); *p* < 0.001	Established adjuvant immunotherapy for high-risk cSCC	[33]
Squamous NSCLC	CheckMate 017	III	Previously treated metastatic	Nivolumab 3 mg/kg IV, Every 2 wk	Docetaxel 75 mg/m^2^ IV, Every 3 wk	OS; PFS	Median OS 9.2 vs. 6.0 mo; HR 0.59 (95% CI: 0.44–0.79); *p* < 0.001 Median PFS 3.5 vs. 2.8 mo; HR 0.62 (95% CI, 0.47–0.81); *p* < 0.001	First PD-1 survival benefit in squamous NSCLC	[34]
Squamous NSCLC	KEYNOTE-407	III	First-line metastatic	Pembrolizumab + carboplatin/taxane Pembrolizumab 200 mg IV every 3 wk + Carboplatin (AUC 6) + Paclitaxel (200 mg/m^2^ every 3 wk) or nab-Paclitaxel (100 mg/m^2^ wk) for 4 cycles Pembrolizumab maintenance	Chemotherapy Saline placebo + Carboplatin + Paclitaxel or nab-Paclitaxel for 4 cycles	OS; PFS	Median OS 15.9 vs. 11.3 mo; HR 0.64 (95% CI, 0.49–0.85); *p* < 0.001 Median PFS:6.4 vs. 4.8 mo; HR 0.56 (95% CI, 0.45–0.70); *p* < 0.001	Established chemo-immunotherapy standard	[35]
Squamous NSCLC	PACIFIC	III	Unresectable stage III	Durvalumab after CRT Durvalumab 10 mg/kg IV every 2 wk for up to 12 mo	Placebo Placebo every 2 wk up to 12 mo	PFS	Median PFS 16.8 vs. 5.6 mo; HR 0.52 (95% CI, 0.42–0.65); *p* < 0.001	Established consolidation immunotherapy after CRT	[36]
Squamous NSCLC	CheckMate 9LA	III	First-line metastatic	Nivolumab + ipilimumab + 2 cycles chemotherapy Nivolumab 360 mg IV every 3 wk + Ipilimumab 1 mg/kg IV every 6 wk + Platinum-doublet chemotherapy (2 cycles)	Chemotherapy Platinum-doublet chemotherapy alone (4 cycles)	OS	Nivolumab + ipilimumab + 2 cycles of chemotherapy vs. 4 cycles of chemotherapy: median OS 15.6 vs. 10.9 mo; HR 0.66 (95% CI, 0.55–0.80) Preplanned interim analysis: median OS 14.1 vs. 10.7 mo; HR 0.69 (96.71% CI, 0.55–0.87); *p* = 0.00065	Validated dual checkpoint blockade strategy	[37]
Squamous NSCLC	IMpower131	III	First-line metastatic	Atezolizumab + carboplatin/nab-paclitaxel Atezolizumab 1200 mg IV every 3 wk + Carboplatin (AUC 6) + nab-Paclitaxel (100 mg/m^2^ wk) for 4–6 cycles → Atezolizumab maintenance	Chemotherapy Carboplatin + nab-Paclitaxel alone for 4–6 cycles	PFS; OS	PFS 6.3 vs. 5.6 mo; HR 0.71 (95% CI 0.60–0.85; *p* = 0.0001 Median OS 14.2 vs. 13.5 mo; HR 0.88 (95% CI, 0.73–1.05); *p* = 0.16	Demonstrated activity of PD-L1 blockade in squamous NSCLC	[38]
ESCC	ATTRACTION-3	III	Previously treated advanced	Nivolumab	Chemotherapy	OS	Median OS 10.9 vs. 8.4 mo; HR 0.77 (95% CI, 0.62–0.96); *p* = 0.019	First PD-1 inhibitor to improve OS in ESCC	[39]
ESCC	KEYNOTE-590	III	First-line advanced/metastatic	Pembrolizumab + cisplatin/5-FU Pembrolizumab 200 mg IV every 3 wk (up to 35 cycles) + 5-FU (800 mg/m^2^/day Days 1–5 every 3 wk) + Cisplatin (80 mg/m^2^ Day 1 every 3 wk for ≤6 cycles)	Chemotherapy Placebo + 5-FU+ Cisplatin	OS	In CPS ≥ 10: Median OS 13.9 vs. 8.8 mo; HR 0.57 (95% CI, 0.43–0.75); *p* < 0.0001 Median PFS 7.5 vs. 5.5 mo; HR 0.51 (95% CI, 0.41–0.65); *p* < 0.0001	Established chemo-immunotherapy as first-line standard	[40]
ESCC	CheckMate 648	III	First-line advanced/metastatic	Nivolumab + chemotherapy Nivolumab 240 mg IV every 2 wk+ Fluorouracil (800 mg/m^2^/day Days 1–5 every 4 wk) + Cisplatin (80 mg/m^2^ Day 1 every 4 wk) or nivolumab + ipilimumab Nivolumab 3 mg/kg IV every 2 wk + Ipilimumab 1 mg/kg IV every 6 wk	Chemotherapy alone Fluorouracil + Cisplatin every 3 wk	OS; PFS	Nivolumab + chemotherapy vs. chemotherapy PD-L1 ≥ 1% population: median OS 15.4 vs. 9.1 mo; HR 0.54 (99.5% CI, 0.37–0.80); *p* < 0.001. PD-L1 ≥ 1% population: PFS HR 0.65 (98.5% CI, 0.46–0.92); *p* = 0.002. Median PFS was not reported in the provided text. Nivolumab + ipilimumab vs. chemotherapy PD-L1 ≥ 1% population: median OS 13.7 vs. 9.1 mo; HR 0.64 (98.6% CI, 0.46–0.90); *p* = 0.001. PD-L1 ≥ 1% population: no significant PFS improvement	Validated both chemo-immunotherapy and dual checkpoint blockade approaches	[39]

Abbreviations: 5-FU, 5-fluorouracil; AUC, area under the curve; CI, confidence interval; CPS, combined positive score; CRT, chemoradiotherapy; cSCC, cutaneous squamous cell carcinoma; DCR, disease control rate; DFS, disease-free survival; DOR, duration of response; EFS, event-free survival; ESCC, esophageal squamous cell carcinoma; HNSCC, head and neck squamous cell carcinoma; HR, hazard ratio; IPI, ipilimumab; IV, intravenous; mo, months; MPR, major pathologic response; NA, not available; Nivo, nivolumab; NSCLC, non-small-cell lung cancer; ORR, objective response rate; OS, overall survival; PD-1, programmed cell death protein 1; PD-L1, programmed death-ligand 1; PFS, progression-free survival; PPR, partial pathological response; pRR, pathological response rate; R/M, recurrent and/or metastatic; SOC, standard of care; wk, week; yr, year.

## Data Availability

No new data were created or analyzed in this study. Data sharing is not applicable.
